# Predictors of Voucher Redemption in Produce Prescription Programs in a Primarily Low-Income Population With Type 2 Diabetes

**DOI:** 10.5888/pcd23.250441

**Published:** 2026-07-02

**Authors:** Tolulope V. Adebile, Kayla Rutt, Christina Scartozzi, William A. Calo, Jennifer L. Kraschnewski, Kelseanna Hollis-Hansen, Susan Veldheer

**Affiliations:** 1 Department of Public Health Sciences, Pennsylvania State College of Medicine, Hershey, Pennsylvania; 2 Department of Family and Community Medicine, Pennsylvania State College of Medicine, Hershey, Pennsylvania; 3 Department of Family and Community Medicine, Pennsylvania State Health St. Joseph Medical Center, Reading, Pennsylvania; 4 Department of Medicine, Pennsylvania State College of Medicine, Hershey, Pennsylvania; 5 Friedman School of Nutrition Science and Policy, Tufts University, Boston, Massachusetts

## Abstract

**Introduction:**

Food as Medicine initiatives, including produce prescription (PRx) programs, provide incentives (ie, vouchers) for participants to purchase free or reduced-cost produce. Voucher redemption is central to PRx effectiveness, yet predictors of redemption are poorly characterized. Prior evaluations often excluded nonusers, potentially overestimating effects. We applied an intent-to-treat (ITT) approach to examine the association between baseline characteristics and voucher redemption rates (VRRs) in a PRx program among primarily low-income Hispanic/Latino adults with type 2 diabetes.

**Methods:**

In this single-arm, pre–post study, 154 participants were enrolled in a 7-month PRx program offering up to $90 in fruit and vegetable vouchers for attending monthly nutrition education sessions. Binomial logistic regression assessed predictors of ITT-VRRs, including transportation access, baseline fruit and vegetable intake, and knowledge of produce preparation.

**Results:**

The sample was majority female (66.2%), Hispanic/Latino (83.1%), and low income (using public health insurance [88.9%] or food insecure [76.0%]). Most participants shared purchased produce with others in the household (61.2%). The ITT-VRR was 65.8%, versus 84.3% in per-protocol analysis. Higher odds of ITT-VRR were associated with transportation access (personal car [odds ratio, 2.28; 95% CI, 1.94–2.67]), while little/no self-reported knowledge of how to prepare fruit and vegetables was associated with lower odds (odds ratio, 0.58; 95% CI, 0.52–0.63).

**Conclusion:**

Transportation access and baseline knowledge of how to prepare fruit and vegetables were associated with voucher redemption, suggesting vouchers alone may not sustain engagement or dietary change. To enhance program equity and effectiveness, future PRx programs may consider pairing vouchers with strategies addressing transportation challenges and supporting fruit and vegetable preparation–related capability.

**Summary**

**What is already known on this topic?**

Produce (fruit and vegetable) prescription programs can improve diet and health outcomes of people with type 2 diabetes, yet voucher redemption rates (VRRs) are low. Studies have identified factors associated with voucher redemption, but most analyses are limited in scope.

**What is added by this report?**

We used an intent-to-treat (ITT) approach to examine associations between baseline characteristics and VRRs in a produce prescription program. ITT-VRRs differed substantially from per-protocol rates and were predicted by access to transportation and baseline knowledge of produce preparation.

**What are the implications for public health practice?**

Produce prescription programs may be more effective when financial incentives are paired with strategies that address transportation barriers and support produce preparation–related capability.

## Introduction

Low fruit and vegetable consumption is a major public health concern in the US, contributing substantially to the burden of diet-related chronic diseases such as type 2 diabetes (1,2). Despite long-standing dietary guidelines emphasizing fruit and vegetable intake, only 10% to 12% of US adults meet daily recommendations, with intake particularly low among populations with lower income (2). Economic constraints, limited access to fresh produce, and a lack of cooking skills affect healthy eating patterns, thereby perpetuating health disparities (3–5).

To address these issues and improve health outcomes, various Food as Medicine initiatives, including produce prescription (PRx) programs, have garnered recent attention. PRx programs provide financial incentives, such as coupons, prepaid cards, credits, vouchers, or direct distribution to facilitate free or subsidized access to fruit and vegetables for people with diet-related chronic diseases like type 2 diabetes (1,6). These programs may lead to improvements in dietary behaviors, body mass index (BMI; calculated as weight in kilograms divided by height in meters squared), blood pressure, and reductions in glycated hemoglobin A_1c_ (HbA_1c_, ranging from 0.1% to 1.4%) among people with diabetes (7–14).

However, the effectiveness of PRx programs requires participants to redeem the incentives provided, with emerging evidence demonstrating an association between voucher redemption and improved health outcomes (7,11). While some studies report a clear linear relationship between higher redemption rates and greater reductions in HbA_1c_ (13), others reveal more nuanced patterns, with health improvements observed even at lower levels of voucher use (0% to <40%) (11). This variability highlights the importance of understanding the baseline characteristics of people who do and do not redeem vouchers to inform program design and improve overall effectiveness.

Although many factors have been identified as significant predictors of voucher redemption (eg, sex, race, Spanish language fluency, total voucher amount, current Supplemental Nutrition Assistance Program [SNAP] participation, number of shopping locations), few studies describe how participants who dropped out of the intervention (ie, nonusers) were handled in analyses or how findings may be generalized more broadly (13,15). Analytically, nonusers can be treated in 2 ways. With an intent-to-treat (ITT) approach, all people who are enrolled are included in the analysis, regardless of their program use. Conversely, a per-protocol analysis includes only participants who used vouchers and were active at the end of the study. Differences in analytic approach can result in potential attrition bias or misrepresentation of program use. For example, in a prior evaluation among primarily Hispanic/Latino low-income populations with type 2 diabetes, average redemption rates increased from 51.5% under an ITT approach to 85.6% under a per-protocol analysis when nonusers were excluded (13). Together, these findings highlight the need to include an ITT approach in PRx programs that will allow for detailed understanding of voucher use and program outcomes.

Given that voucher redemption is a central element in the behavioral pathway to fruit and vegetable intake and in effective programs, we investigated the factors that predict voucher redemption among a primarily low-income population enrolled in a PRx program. By using an ITT approach that included all participants, regardless of their voucher redemption status, our primary aim was to examine the association between baseline characteristics (eg, sociodemographic characteristics, transportation access, nutrition or preparation knowledge, BMI, HbA_1c_) and voucher redemption rates (VRRs). As a secondary objective, we examined the program’s effects on behavioral and health outcomes and the proportion of participants who shared the produce they purchased with others in their household. Results from this study will advance the evidence base for the effectiveness of PRx programs and can guide policy development, particularly for people with diabetes. Additionally, results will help identify key factors to direct future program implementation, enhance use, and ultimately improve health outcomes.

## Methods

### Study design and population

We used data from a single arm, pre–post design study that occurred from December 2021 through December 2023 at Penn State Health St. Joseph Medical Center in Reading, Pennsylvania, a primarily low-income community (70.8% Hispanic and 28.7% in poverty) (16). Eligible participants were adults (aged ≥18 y) who were receiving primary care at the clinic, were diagnosed with type 2 diabetes, with HbA_1c_ of ≥7.0 and a BMI of ≥25 kg/m². We excluded people unable or unwilling to provide informed consent or people receiving care outside the clinic. Clinicians and staff prescreened participants with scheduled clinic appointments to identify eligible participants.

During a clinic visit, eligible participants were informed about the potential benefits of dietary modifications for glucose control, particularly increased fruit and vegetable consumption. Health care providers, including a community health worker (CHW), a diabetes educator, program staff, and resident or attending physicians introduced the program and distributed informational brochures.

At the end of each participant’s visit, the CHW and program staff provided program details, obtained written informed consent, and issued an initial $28 paper voucher packet with a list of participating vendors. Participants also completed baseline surveys and scheduled their first group-based nutrition education session.

This study was approved by the Penn State College of Medicine Institutional Review Board (protocol: AFFILVEGGIERX). We used the Research Electronic Data Capture (REDCap) online database (17) to collect and manage data.

### Intervention: PRx program

Participants received additional vouchers upon attending monthly nutrition education sessions at the clinic. The number of vouchers provided was based on household size, and vouchers could be redeemed at participating vendors, including 7 local farmers markets/stands, 5 corner stores, and a single grocery store. Monthly voucher amounts were $18 for a household of 1 (participant only), $36 for 2, $54 for 3, $72 for 4, and $90 for 5 or more. Including baseline vouchers, total allotments were $136 for a household of 1 (participant only), $244 for 2, $352 for 3, $460 for 4, and $568 for a household of 5 or more. Each voucher had a distinct, sequential numeric code recorded at distribution and redemption to track use. All vouchers were valid through December 2024.

The 6 monthly, in-person, 1-hour, group-based nutrition education sessions were led by a bilingual (English and Spanish) CHW and based on the American Diabetes Association’s *Life with Diabetes* curriculum (18). Sessions aimed to build participants’ confidence in managing diabetes through practical lifestyle strategies, including nutrition knowledge and interactive skill building on topics such as understanding complex versus refined carbohydrates, meal planning and portion control, reading nutrition labels, produce-focused cooking skills, and choosing heart-healthy fats.

### Primary and secondary study outcomes

The primary outcome was ITT-VRR, calculated for each participant and the overall study population as the number of vouchers redeemed divided by the number of vouchers distributed. To calculate the ITT-VRR for nonusers, we assumed that all allocated vouchers were distributed but none were redeemed.

Secondary outcomes included changes in fruit and vegetable intake, BMI, and HbA_1c_ from baseline (preintervention) to 7-month follow-up (postintervention). We used 6 National Health and Nutrition Examination Survey (NHANES) dietary screener questions to evaluate fruit and vegetable intake: for example, “(During the past month), how often did you eat fruit? Do not include juices” and “(During the past month), how often did you eat a green leafy or lettuce salad, with or without other vegetables?”). Responses (“never” to “4 or more times per day”) were standardized to daily equivalents and summed to estimate total intake.

The CHW measured height and weight at baseline and follow-up by using the Pro Doc PD300 scale (Detecto) for weight and the 2011 Seca 216 (Seca Corp) wall-mounted stadiometer for height; calculated BMI as weight in kilograms divided by height in meters squared; and used a point-of-care analyzer, the Alere Afinion AS100 analyzer (Abbott Diagnostics Technologies AS), to assess HbA_1c_.

### Covariates

Baseline covariates included self-reported age; biological sex (female, male); Spanish-speaking only (yes, no); education (less than high school diploma, high school diploma/General Educational Development, more than high school diploma); health insurance (public [Medicaid, Medicare], private, none); total household size (number of people, including children); food insecurity (yes, no); shopping location for groceries (convenience store, supermarket/large food retailer, small grocery store); transportation method to shopping location (personal car, public bus, someone else’s car, walking, other); current SNAP beneficiary; numbers of markets used; produce sharing within household, BMI; and HbA_1c_.

We used 2 items from the US Department of Agriculture 6-item Short Form (19) to assess food insecurity, with participants classified as food insecure if they responded “sometimes” or “often true” to either of the following: “The food didn’t last, and we didn’t have money to get more” and/or “We couldn’t afford to eat balanced meals.”

Other measures collected at baseline and during the program by the study team included fruit and vegetable intake, awareness of participating retailers or farmers markets and the importance of fruit and vegetables in the family’s diet, knowledge of how to prepare fruit and vegetables, number of nutrition education sessions attended, and completion of a postintervention survey at program completion. Questions on knowledge of how to prepare fruit and vegetables and the awareness of participating retailers or farmers markets and of the importance of fruit and vegetables were adapted from the Wholesome Wave Georgia FVRx Toolkit (20). We combined these responses into to “little/none” and “some/a lot” to improve estimate stability and interpretability.

### Statistical analysis

To summarize characteristics of participants, we used frequencies and percentages for categorical variables and means, medians, IQRs, ranges, and SDs for continuous variables. We used the Fisher exact test for paired data to assess differences in knowledge of participating markets, produce preparation, and importance of fruit and vegetables preintervention versus postintervention.

To examine the association between baseline characteristics and ITT-VRR, we used binomial regression, incorporating age, biological sex, Spanish-speaking only, health insurance, food insecurity, household size, education, transportation method, shopping location, awareness of participating retailers or farmers markets, fruit and vegetable intake, knowledge of how to prepare fruit and vegetables, awareness of the importance of fruit and vegetables, BMI, and HbA_1c_. The model accounted for the bounded (0–1) nature of VRR, producing odds ratios (ORs) and 95% Wald CIs to quantify associations. These estimates help identify which factors were positively or negatively associated with ITT-VRR.

To evaluate the robustness of associations, we conducted a sensitivity analysis using a per-protocol approach (actual VRR), without assumptions regarding voucher distribution or redemption. Additional sensitivity analyses were performed among program completers to assess the robustness of ITT-VRR associations and to examine whether results were consistent when accounting for intervention-related factors. In this analysis, we included changes from baseline to follow-up (ie, changes in awareness of retailers, HbA_1c_, BMI, fruit and vegetable intake, produce preparation knowledge, and importance of fruit and vegetables).

We used paired *t* tests to test preintervention and postintervention changes in BMI, HbA_1c_, and fruit and vegetable intake. Unless otherwise stated, analyses were 2-sided, with *P* <.05 considered significant, and we used SAS version 9.4 (SAS Institute Inc) for all analyses.

## Results

Most participants were female (66.2%) and identified as Spanish-speaking only (56.5%) (Table 1). Most had not completed high school (61.7%), used public health insurance (88.9%), were food insecure (76.0%), had a mean (SD) household size of 3.2 (1.9) people, and reported sharing the produce they purchased with at least 1 other person in their household (61.2%).

**Table 1:** Characteristics of Participants (N = 154) in a Study on Predictors of Voucher Redemption in Produce Prescription Programs, Reading, Pennsylvania, December 2021–December 2023^a^

Characteristic	Value
**Program completion, no. (%)**	108 (70.1)
**Age, mean (SD), y**	57.8 (11.8)
**Baseline body mass index, mean (SD)**	34.3 (7.6)
**Baseline hemoglobin A_1c_ mean % (SD)**	9.5 (2.0)
**Female sex, no. (%)**	102 (66.2)
**Mean no. of people in household (SD) [range]**	3.2 (1.9) [1–11]
**No. of people in household, including children, no. (%)**	
1	26 (16.9)
2–4	90 (58.4)
≥5	38 (24.7)
**Race, no. (%)**	
African American or Black	22 (14.3)
American Indian/Alaska Native	1 (0.7)
White	78 (50.7)
Other	53 (34.4)
**Hispanic/Latino/Spanish, no. (%)**	128 (83.1)
**Education level, no. (%)**	
Less than high school diploma	95 (61.7)
High school diploma or General Educational Development	33 (21.4)
More than high school diploma	26 (16.7)
**Spanish-speaking only**	87 (56.5)
**Health insurance status, no. (%)**	
Public	136 (88.9)
Private	11 (7.2)
None	6 (3.9)
**Current SNAP beneficiary**	99 (64.3)
**Has food insecurity**	117 (76.0)
**No. of markets used, no. (%)**	
0	28 (18.2)
1	112 (72.7)
≥2	14 (9.1)
**No. of people in household with whom produce was shared, no. (%)**	
0	52 (38.8)
≥1 Other	82 (61.2)
**Transportation method to primary shopping location for groceries, no. (%)**	
Personal car	38 (34.9)
Someone else’s car	53 (48.6)
Public bus	7 (6.4)
Walking	11 (10.1)
**Primary shopping location for groceries, no. (%)**	
Convenience/small grocery store	10 (6.5)
Supermarket/large food retailer	144 (93.5)

Abbreviation: SNAP, Supplemental Nutrition Assistance Program.

a Study participants were adults with type 2 diabetes, HbA_1c_ of ≥7.0 and body mass index ≥25 kg/m^2^, enrolled in a 7-month fruit and vegetable prescription program from December 2021 through December 2023 at Penn State Health St. Joseph Medical Center in Reading, Pennsylvania. Missing values for race (n = 26), Hispanic/Latino/Spanish (n = 9), health insurance (n = 1), food insecurity (n = 1), current SNAP beneficiary (n = 1), transportation method (n = 45), and produce sharing (n = 20).

The overall ITT-VRR for the study population (N = 154) was 65.8% (17,790 redeemed/27,050 distributed), and the VRR from the per-protocol analysis (actual-VRR [n = 153]) was 84.3% (17,971 redeemed/21,306 distributed). Individual ITT-VRR ranged from 0 vouchers redeemed (n = 19, 12.3% of sample) to 100% of vouchers redeemed (n = 14, 9.1% of sample). Among the 154 participants, 108 (70.1%) completed the postintervention survey, with a mean (SD) ITT-VRR of 63.3% (40.0) and mean (SD) actual VRR of 76% (33.5). On average, participants attended 5.3 (SD, 1.5) nutrition sessions and received a median total voucher amount of $352. Most reported using either someone else’s car (48.6%) or their personal car (34.9%) to reach their shopping location, which was mostly at supermarkets/large food retailers (93.5%).

We found a steady decline in session attendance throughout the program (Figure). The actual VRR was 81.3% on enrollment, peaked at 92.7% in session 2, and subsequently declined to 75.4% by session 6. The ITT-VRR followed a similar pattern, decreasing from 78.8% at baseline to 71.4% in session 1, increasing slightly to 73.1% in month 2, and then declining consistently to 55.3% by session 6.

**Figure:**
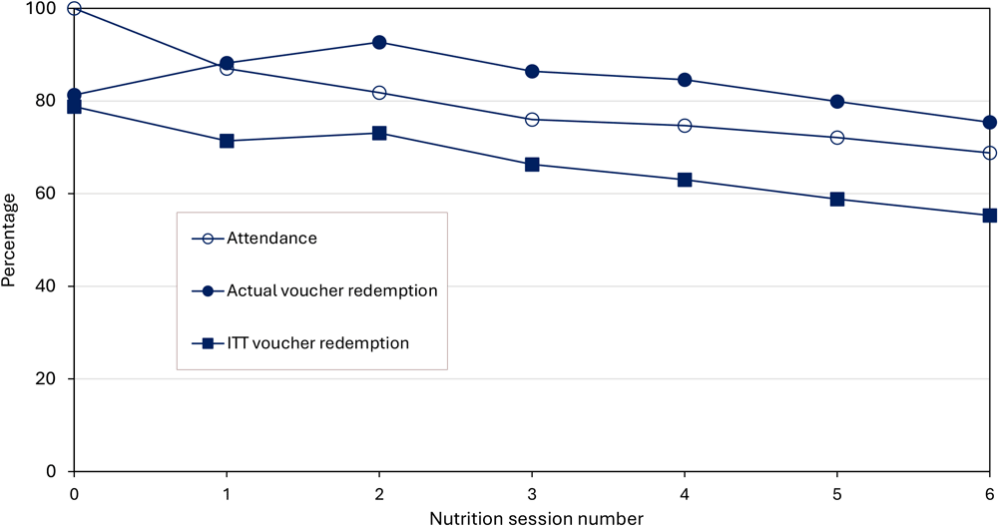
Voucher redemption rate (ITT and actual) and attendance rate, by nutrition session number, among participants (n = 154). Participants were adults with type 2 diabetes, HbA_1c_ of ≥7.0 and body mass index ≥25 kg/m^2^ enrolled in a 7-month fruit and vegetable prescription program from December 2021 through December 2023 at Penn State Health St. Joseph Medical Center in Reading, Pennsylvania. The program hosted 6 nutrition sessions, session 1 through session 6.

**Table:** 

Nutrition session number	Attendance	Actual voucher redemption	ITT voucher redemption
0	100.0	81.3	78.8
1	87.0	88.2	71.4
2	81.8	92.7	73.1
3	76.0	86.4	66.3
4	74.7	84.6	63.0
5	72.1	79.9	58.8
6	68.8	75.4	55.3

A significant increase in the odds of voucher redemption was associated with access to a personal car (vs walking, OR, 2.28; 95% CI, 1.94–2.67), higher fruit and vegetable intake (OR, 1.30; 95% CI, 1.28–1.33), and little/no baseline awareness of the importance of fruit and vegetables (vs some/a lot, OR, 1.66; 95% CI, 1.49–1.84) (Table 2). Older participants, females (vs males), Spanish-speaking only (vs no), higher education, convenience/small grocery store users, other transportation, and those from larger households were also significantly and positively associated with higher rates of voucher redemption.

**Table 2:** Predictors of Voucher Redemption Rate Among Participants (N = 154) in a Study on Predictors of Voucher Redemption in Produce Prescription Programs, Reading, Pennsylvania, December 2021–December 2023^a^

Covariate	Odds ratio (95% Cl)
**Age, y**	1.02 (1.02–1.02)
**Sex**	
Female	1.65 (1.52–1.80)
Male	1 [Reference]
**Spanish-speaking only**	
Yes	1.11 (1.01–1.22)
No	1 [Reference]
**Health insurance status**	
Public	0.80 (0.66–0.98)
Private	0.87 (0.68–1.11)
None	1 [Reference]
**Food insecurity**	
Yes	1.09 (0.99–1.20)
No	1 [Reference]
**No. of people in household, including children**	
2–4	2.20 (1.98–2.45)
≥5	2.10 (1.83–2.40)
1	1 [Reference]
**Education**	
High school diploma/General Educational Development	1.74 (1.56–1.92)
More than high school	1.92 (1.70–2.17)
Less than high school diploma	1 [Reference]
**Transportation method to shopping location for groceries**	
Personal car	2.28 (1.94–2.67)
Public bus	0.72 (0.59–0.88)
Someone else’s car	0.99 (0.85–1.15)
Other	2.12 (1.44–3.10)
Walking	1 [Reference]
**Primary shopping location for groceries**	
Convenience/small grocery store	1.24 (1.04–1.47)
Supermarket/large food retailer	1 [Reference]
**Awareness of participating retailer/farmers markets**	
Little/none	0.39 (0.35–0.43)
Some/a lot	1 [Reference]
**Preintervention fruit and vegetable intake**	1.30 (1.28–1.33)
**Knowledge of how to prepare fruit and vegetables**	
Little/none	0.58 (0.52–0.63)
Some/a lot	1 [Reference]
**Awareness of the importance of fruit and vegetables**	
Little/none	1.66 (1.49–1. 84)
Some/a lot	1 [Reference]
**Body mass index**	0.83 (0.80–0.87)
**Hemoglobin A_1c_**	0.80 (0.77–0.83)

a Study participants were adults with type 2 diabetes, HbA_1c_ of ≥7.0 and body mass index ≥25 kg/m^2^, enrolled in a 7-month fruit and vegetable prescription program from December 2021 through December 2023 at Penn State Health St. Joseph Medical Center in Reading, Pennsylvania. Binomial logistic regression was used; 2 participants were excluded due to missing values for the response or explanatory variables.

Conversely, lower odds of ITT-VRR were associated with little/no baseline knowledge of how to prepare fruit and vegetables (vs some/a lot, OR, 0.58; 95% CI, 0.52–0.63), little/no baseline awareness of participating retailers or farmers markets (vs some/a lot of baseline awareness, OR, 0.39; 95% CI, 0.35–0.43), using public health insurance, using public transportation, and having higher BMI or HbA_1c_ levels.

Sensitivity analyses using the actual-VRR model and among program completers yielded broadly consistent findings. Among program completers, public insurance was associated with significantly higher ITT-VRR, whereas shopping at convenience or small grocery stores was associated with lower ITT-VRR. Additionally, when accounting for intervention-related factors, decline or no change in fruit and vegetable preparation knowledge was significantly associated with higher ITT-VRR.

From preintervention to postintervention, we found a significant decrease in HbA_1c_, a nonsignificant decline in BMI, and a slight nonsignificant increase in fruit and vegetable intake both with and without potatoes (Table 3). We found no significant changes in participant awareness of participating retailers or farmers markets, knowledge of how to prepare fruit and vegetables, and knowledge of the importance of fruit and vegetables (Table 4).

**Table 3:** Pre–Post Mean Changes in Fruit and Vegetable Intake and Biometric Results Among a Subsample of Participants (n = 108) in a Study on Predictors of Voucher Redemption in Produce Prescription Programs, Reading, Pennsylvania, December 2021–December 2023^a^

Category	Preintervention	Postintervention	Change	*P* value^b^
**Fruit and vegetable intake, mean (SD), times per day**				
Fruit and vegetables with potatoes	3.4 (2.2)	3.5 (2.6)	0.16 (−0.20 to 0.51)	.38
Fruit and vegetables without potatoes	3.0 (2.1)	3.0 (2.0)	0.04 (−0.27 to 0.35)	.81
**Biometrics**				
Body mass index^c^	34.19 (7.73)	34.09 (7.65)	−0.11 (−0.47 to 0.26)	.56
Hemoglobin A_1c_, mean (SD), %	9.31 (1.98)	8.75 (2.05)	−0.56 (−0.83 to −0.29)	<.001

a Study participants were adults with type 2 diabetes, HbA_1c_ of ≥7.0 and body mass index ≥25 kg/m^2^, enrolled in a 7-month fruit and vegetable prescription program from December 2021 through December 2023 at Penn State Health St. Joseph Medical Center in Reading, Pennsylvania. Only 108 study participants had complete pre–post intervention biometric data.

b Determined by using paired *t* tests.

c Calculated as weight in kilograms divided by height in meters squared.

**Table 4:** Pre–Post Differences in Knowledge of Participating Markets and the Preparation and Importance of Fruit and Vegetables Among a Subsample of Participants (n = 109) in a Study on Predictors of Voucher Redemption in Produce Prescription Programs, Reading, Pennsylvania, December 2021–December 2023^a^

How much do you feel you know about the following . . .	Preintervention, no. (%)	Postintervention, no. (%)	*P* value^b^
**Retailers or farmers markets that participate in this program**			
Little/none	72 (66.1)	40 (36.7)	.30
Some/a lot	37 (33.9)	69 (63.3)	
**How to prepare fresh fruit and vegetables**			
Little/none	45 (41.5)	16 (14.7)	.58
Some/a lot	64 (58.7)	93 (85.3)	
**The importance of fruit and vegetables in your family’s diet**			
Little/none	38 (34.9)	17 (15.6)	.10
Some/ a lot	71 (65.1)	92 (84.4)	

a Study participants were adults with type 2 diabetes, HbA_1c_ of ≥7.0 and body mass index ≥25 kg/m^2^, enrolled in a 7-month fruit and vegetable prescription program from December 2021 through December 2023 at Penn State Health St. Joseph Medical Center in Reading, Pennsylvania. Only 109 study participants had complete pre–post intervention nutrition-related data.

b Determined by using Fisher exact binomial tests for paired data.

## Discussion

This single-arm, pre–post study of a PRx program among primarily low-income Hispanic/Latino adults with type 2 diabetes used an ITT analytic approach to examine how baseline participant characteristics influenced voucher redemption, a key factor in the behavioral pathway to improving health outcomes in PRx programs. This study highlights 3 key findings. First, VRR varied substantially by analytic approach, with the ITT method yielding a lower redemption rate (ITT-VRR = 65.8%) than the per-protocol approach (actual VRR = 84.3%). Second, having access to a personal car emerged as a strong predictor of voucher redemption in this sample, and third, little/no baseline knowledge of how to prepare fruit and vegetables was associated with lower odds of ITT-VRR, suggesting that food preparation–related capability may influence PRx program engagement.

The VRR was 18.5 percentage points higher in the per-protocol analysis than in the ITT analysis, which assumed that unredeemed vouchers among nonusers were distributed but not used. Previous PRx evaluations demonstrated similar discrepancies (13), underscoring the importance of consistent reporting practices. Our overall ITT-VRR (65.8%), although comparable to rates observed in other PRx programs (>70%), was lower (15,21). Together, these findings reflect contextual variability across programs and emphasize the value of reporting both ITT and per-protocol rates to provide a more comprehensive understanding of program performance and participant engagement.

Access to transportation emerged as a strong predictor of voucher redemption in this sample, particularly access to a personal car. This finding aligns with prior research identifying transportation as a barrier to redemption in PRx programs and reinforces its continued relevance for program engagement (22–24). Prior PRx and nutrition incentive initiatives similarly recognized these challenges and explored strategies such as mobile markets (eg, Veggie Van) to improve access (25). Our results add to this body of evidence by suggesting that transportation may influence voucher use in a real-world PRx program context.

Finally, the association between prior knowledge of fruit and vegetable preparation and ITT-VRR may reflect the role of perceived familiarity with preparing fruit and vegetables. Similar patterns have been observed in prior work; for example, Wetherill et al reported that greater knowledge of vegetable preparation was associated with higher redemption rates among lower-income families (26). Although we assessed preparation knowledge by using a single self-reported question rather than a comprehensive measure of food skills, participants reporting greater knowledge of fruit and vegetable preparation at baseline demonstrated higher voucher redemption. This finding suggests that baseline familiarity with or confidence in preparing fruit and vegetables may influence participants’ likelihood of engaging in PRx programs, redeeming vouchers, or changing their fruit and vegetable intake (27–29). It also highlights broader behavioral considerations for PRx engagement. Although vouchers increase the opportunity to purchase high-quality fruit and vegetables, participants may differ in psychological capability (eg, familiarity with preparation) and motivation to fully benefit from them. The Capability, Opportunity, Motivation–Behavior model provides a useful lens for understanding these dynamics (30,31). Together, these findings suggest that preparation-related capability may influence engagement and that future PRx studies may benefit from further exploring how baseline skill levels affect program outcomes.

In addition, the sharing of produce within households in this study suggests potential social intervention spillover effects, whereby PRx programs may influence family food environments and dietary habits beyond enrolled participants. Designing PRx programs that intentionally engage family members through household-based education or cooking activities may amplify these social pathways, enhance sustained engagement, and broaden program impact. Framing PRx programs through the Capability, Opportunity, Motivation–Behavior model or similar models may help implementers target behavioral levers and ultimately improve voucher redemption, dietary intake, and program effectiveness (27,32).

### Strengths and limitations

This study has several notable strengths, including the rigorous application of an ITT approach to systematically assess predictors of voucher redemption. To the best of our knowledge, it is one of the first to do so in the context of PRx programs, and we believe it represents a strong model for programmatic and policy-relevant program evaluation. In addition, we included program outcomes as well as baseline participant characteristics and nutrition-related behaviors. These variables allow us to explore intervention components that may be necessary for effective PRx programs that are aligned with evidence-based behavioral theory. We were also able to apply behavioral theory, such as the Capability, Opportunity, Motivation–Behavior model, to contextualize our findings, which strengthens the interpretability of our results and highlights the importance of applying behavioral theory to enhance program engagement. Lastly, the detailed accounting of voucher distribution and redemption at the participant level enhanced analytic precision and allowed for a more nuanced exploration of individual-level engagement patterns, offering valuable insight for future program design and evaluation.

Some limitations warrant consideration. First, the observational design limits causal inference. Second, although we used standardized instruments where possible (eg, US Department of Agriculture food security module), behavioral and knowledge-related measures were based on measures that are not comprehensive, validated food skills instruments but rather those recommended in a toolkit that was widely used at the time the study was designed. Therefore, findings related to preparation knowledge should be interpreted cautiously. Future PRx evaluations may benefit from incorporating more rigorously validated food skills measures such as those described by Lavelle and colleagues (4).

Voucher redemption rates varied widely across participants, indicating substantial heterogeneity in program engagement; this variability may limit the precision of estimated predictors. Sensitivity analyses using actual VRR and among program completers produced broadly similar findings, with some differences among completers, suggesting potential influence of program participation, ceiling effects, or residual confounding. Results should therefore be interpreted as exploratory and hypothesis-generating rather than as definitive causal relationships.

Clinical and public health professionals might consider 3 actionable priorities for the funding, design, and implementation of PRx programs. First, transportation access may be a critical structural challenge to program engagement at baseline, particularly in small cities and suburban or rural areas with limited public transit. Complementary strategies such as colocating voucher redemption sites near transit routes, deploying mobile markets, or offering online ordering and home delivery options may increase access, promote equitable program reach, and support meaningful dietary improvement.

Second, PRx programs may benefit from tailoring fruit and vegetable education to participants’ baseline skill levels. This may be particularly important given the elimination in 2025 of SNAP-Ed funding, which had historically supported nutrition education and development of food preparation skills among low-income populations. Vouchers increase an individual’s *opportunity* to purchase fruit and vegetables, but additional intervention components may be needed to enhance *capability* and *motivation.* Integrating culturally relevant meal plans; recipes; and hands-on, step-by-step cooking sessions delivered in-person or virtually can support participants in using the produce purchased. Given the common practice of household produce sharing, future interventions should also consider family dynamics to reinforce healthy eating habits. This approach aligns with culinary medicine, which integrates nutritional education with experiential skill building to support sustainable dietary changes (33). Recent evidence also emphasizes the need to tailor cooking interventions to participants’ cultural preferences and baseline skill levels, using education, modeling, and enablement as core strategies (34,35).

Third, strengthening evaluation and reporting practices is critical to advancing the evidence base for Food as Medicine initiatives. Consistent reporting of both ITT and per-protocol VRRs will enhance comparability across studies and enhance the rigor of PRx evaluations, providing data-driven guidance for implementation, funding, and policy decisions.

### Conclusion

We used an ITT approach to highlight access to transportation and baseline knowledge of fruit and vegetable preparation as key predictors of PRx voucher redemption. Results suggest that vouchers alone may be insufficient to drive sustained engagement in PRx programs. Future PRx initiatives should consider pairing financial incentives with strategies that address structural barriers and support fruit and vegetable preparation–related capability.
